# Associations of angiogenesis-related proteins with specific prognostic factors, breast cancer subtypes and survival outcome in early-stage breast cancer patients. A Hellenic Cooperative Oncology Group (HeCOG) trial

**DOI:** 10.1371/journal.pone.0200302

**Published:** 2018-07-31

**Authors:** Anna Goussia, Nafsika Simou, Flora Zagouri, Kyriaki Manousou, Georgios Lazaridis, Helen Gogas, Angelos Koutras, Maria Sotiropoulou, George Pentheroudakis, Dimitrios Bafaloukos, Christos Markopoulos, Helen Patsea, Christos Christodoulou, Pavlos Papakostas, Thomas Zaramboukas, Epaminontas Samantas, Paris Kosmidis, Vasileios Venizelos, Charisios Karanikiotis, George Papatsibas, Grigorios Xepapadakis, Konstantine T. Kalogeras, Christina Bamia, Meletios-Athanassios Dimopoulos, Vassiliki Malamou-Mitsi, George Fountzilas, Anna Batistatou

**Affiliations:** 1 Department of Pathology, Ioannina University Hospital, Faculty of Medicine, School of Health Sciences, University of Ioannina, Ioannina, Greece; 2 Department of Clinical Therapeutics, Alexandra Hospital, National and Kapodistrian University of Athens School of Medicine, Athens, Greece; 3 Section of Biostatistics, Hellenic Cooperative Oncology Group, Athens, Greece; 4 Department of Medical Oncology, Papageorgiou Hospital, Aristotle University of Thessaloniki, School of Health Sciences, Faculty of Medicine, Thessaloniki, Greece; 5 First Department of Medicine, Laiko General Hospital, National and Kapodistrian University of Athens School of Medicine, Athens, Greece; 6 Division of Oncology, Department of Medicine, University Hospital, University of Patras Medical School, Patras, Greece; 7 Department of Pathology, Alexandra Hospital, Athens, Greece; 8 Department of Medical Oncology, Ioannina University Hospital, Ioannina, Greece; 9 First Department of Medical Oncology, Metropolitan Hospital, Piraeus, Greece; 10 Second Department of Prop. Surgery, Laiko General Hospital, National and Kapodistrian University of Athens School of Medicine, Athens, Greece; 11 Department of Pathology, IASSO General Hospital, Athens, Greece; 12 Second Department of Medical Oncology, Metropolitan Hospital, Piraeus, Greece; 13 Oncology Unit, Hippokration Hospital, Athens, Greece; 14 Department of Pathology, Aristotle University of Thessaloniki, School of Health Sciences, Faculty of Medicine, Thessaloniki, Greece; 15 Third Department of Medical Oncology, Agii Anargiri Cancer Hospital, Athens, Greece; 16 Second Department of Medical Oncology, Hygeia Hospital, Athens, Greece; 17 Breast Unit, Metropolitan Hospital, Piraeus, Greece; 18 Department of Medical Oncology, 424 Army General Hospital, Thessaloniki, Greece; 19 Oncology Department, University General Hospital of Larissa, Larissa, Greece; 20 Breast Clinic, REA Hospital, Piraeus, Greece; 21 Translational Research Section, Hellenic Cooperative Oncology Group, Athens, Greece; 22 Laboratory of Molecular Oncology, Hellenic Foundation for Cancer Research/Aristotle University of Thessaloniki, Thessaloniki, Greece; 23 Department of Hygiene, Epidemiology and Medical Statistics, National and Kapodistrian University of Athens School of Medicine, Athens, Greece; 24 Aristotle University of Thessaloniki, Thessaloniki, Greece; Children's Hospital Boston, UNITED STATES

## Abstract

Several studies support an important role of angiogenesis in breast cancer growth and metastasis. The main objectives of the study were to investigate the immunohistochemical expression of vascular endothelial growth factor (VEGF) family ligands (VEGF-A and VEGF-C) and receptors (VEGFR1, VEGFR2 and VEGFR3) in breast cancer and their associations with clinicopathological parameters, cancer subtypes/subgroups and patient outcome. Formalin-fixed paraffin-embedded tumor tissue samples were collected from early-stage breast cancer patients treated with anthracycline-based chemotherapy within a randomized trial. Immunohistochemistry was performed on serial 2.5 μm thick tissue sections from tissue microarray blocks. High VEGF-A, VEGF-C, VEGFR1, VEGFR2 and VEGFR3 protein expression was observed in 11.8% (N = 87), 80.8% (N = 585), 28.1% (N = 202), 64.6% (N = 359) and 71.8% (N = 517) of the cases, respectively. Significant associations were observed among all proteins (all p-values <0.05), with the exception of the one between VEGF-C and VEGFR1 (chi-square test, p = 0.15). Tumors with high VEGF-A protein expression, as compared to tumors with low expression were more frequently ER/PgR-negative (33.3% vs. 20.8%, chi-square test, p = 0.009) and HER2-positive (44.8% vs. 20.6%, p<0.001). In addition, tumors with high VEGFR1 expression, were more frequently HER2-positive (32.8% vs. 19.6%, p<0.001), while tumors with high VEGFR3 expression were more frequently ER/PgR-negative (24.9% vs. 17.0%, p = 0.024) and HER2-positive (26.9% vs. 14.8%, p = 0.001). High VEGF-A and VEGF-C protein expression was associated with increased DFS in the entire cohort (HR = 0.57, 95% CI 0.36–0.92, Wald’s p = 0.020 and HR = 0.71, 95% CI 0.52–0.96, p = 0.025, respectively), as well as in specific subtypes/subgroups, such as HER2-positive (VEGF-A, HR = 0.32, 95% CI 0.14–0.74, p = 0.008) and triple-negative (VEGF-C, HR = 0.44, 95% CI 0.21–0.91, p = 0.027) patients. High vs. low VEGFR1 expression was an unfavorable factor for DFS in triple-negative patients (HR = 2.74, 95% CI 1.26–5.98, p = 0.011), whereas the opposite was observed among the ER/PgR-positive patients (HR = 0.69, 95% CI 0.48–0.98, p = 0.041). Regarding OS, high VEGF-C protein expression was associated with increased OS in the entire cohort (HR = 0.64, 95% CI 0.46–0.89, Wald’s p = 0.008), as well as in in specific subtypes/subgroups, such as ER/PgR-negative (HR = 0.37, 95% CI 0.20–0.71, p = 0.003) and triple-negative (HR = 0.42, 95% CI 0.19–0.90, p = 0.026) patients. In conclusion, high expression of angiogenesis-related proteins is associated with adverse clinicopathological parameters in early-stage breast cancer patients and may be surrogate markers of biologically distinct subgroups of ER/PgR-negative or triple-negative tumors with superior outcome. Further validation of our findings in independent cohorts is needed.

## Introduction

Experimental and clinical studies suggest that breast cancer is an angiogenic dependent disease and that angiogenesis plays an important role in tumor development and metastasis[1, 2]. A number of angiogenic factors are expressed by several human tumors, including breast cancer. Among them, the most important are the members of the vascular endothelial growth factor (VEGF) family and their receptors (VEGFRs). The VEGF family consists of five structurally homologous proteins: VEGF-A, -B, -C, -D and -E, with the first three being better characterized in terms of mechanism of action. VEGF-A and -B are considered mainly angiogenic, while VEGF-C mediates lymphangiogenesis. The biological effects of VEGF-A are mediated by two tyrosine kinase receptors: VEGFR1 and VEGFR2. Both receptors are predominantly expressed in vascular endothelial cells[3]. VEGFR2 mediates major growth and permeability actions of VEGF-A[4], whereas VEGFR1 has a weaker signal transducing ability either by acting as a decoy receptor or by suppressing signaling through VEGFR2[5]. VEGF-B forms heterodimers with VEGF-A and has two binding receptors: VEGFR1 and neuropilin-1(3). VEGF-C mediates lymphangiogenesis through binding to VEGFR3 and angiogenesis through binding to VEGFR2[6].

The main prognostic biomarkers in early-stage breast cancer are tumor size, grade, lymph node status, number of positive lymph nodes, estrogen and progesterone receptor (ER, PgR) status and epidermal growth factor receptor 2 (HER2) status[7]. These variables are used to identify patients who are more likely to benefit from hormonal therapy and adjuvant chemotherapy. To improve the therapeutic ratio of breast cancer patients, research efforts focus on the identification of other prognostic and predictive biomarkers. Current evidence indicates that breast carcinoma cells express angiogenic factors at the mRNA or protein level[1, 8–21] and their clinicopathological significance has been studied in both, node-negative and node-positive breast cancers with inconsistent results[8, 10, 13–15, 18, 20–23]. The conflicting findings are in part due to the molecular heterogeneity of the disease, to different antibodies used and to variability in the selected cut-offs. Although the role of VEGF-A is more well studied and in several reports its expression has been correlated with adverse clinicopathological parameters[8, 14], the significance of the other ligands and receptors is yet unclear.

In the present study, we evaluated the immunohistochemical expression of VEGF-A, VEGF-C, VEGFR1, VEGFR2 and VEGFR3 in tumor tissue sections of early-stage breast cancer patients who had participated in a randomized adjuvant chemo-hormonotherapy trial of the Hellenic Cooperative Oncology Group (HeCOG)[24] and correlated it with clinicopathological parameters, cancer subtypes and survival outcome.

## Materials and methods

### Study population

This is translational research study among 1,086 early-stage breast cancer patients, enrolled in a prospective randomized phase III trial (HE10/00) of the Hellenic Cooperative Oncology Group (HeCOG), that was included in the Australian New Zealand Clinical Trials Registry (ANZCTR) and allocated Registration Number ACTRN12609001036202. The clinical protocol was approved by the HeCOG Protocol Review Committee, the Institutional Review Board of the AHEPA University and by the Bioethics Committee of the Aristotle University of Thessaloniki, School of Medicine. All patients signed a written informed consent for the use of their biological material for future research purposes.

The patients were randomized to receive concurrent or dose-dense sequential administration of epirubicin (E) and paclitaxel (T), followed by dose-dense cyclophosphamide/methotrexate/fluorouracil (CMF) (ET-CMF versus E-T-CMF)(24). By study design, all patients were female, with the cumulative doses and the chemotherapy duration being identical in the two arms but dose intensity of epirubicin and paclitaxel being double in the E-T-CMF arm. Prophylactic administration of granulocyte-colony stimulating factor (G-CSF) was administered in all cycles with CMF. Premenopausal women received additional treatment with lutenizing hormone-releasing hormone (LH-RH) analogs for two years. Postmenopausal patients received tamoxifen 20 mg daily for two-three years followed two-three years of daily exemestane 25 mg. Hormonal therapy or radiation therapy was administered after the completion of chemotherapy.

Baseline characteristics and clinical outcomes of this trial have recently been described(24). Tumor size, histological grade and lymph node status were obtained from the pathology report.

### Tumor tissue samples

**Fig 1:**
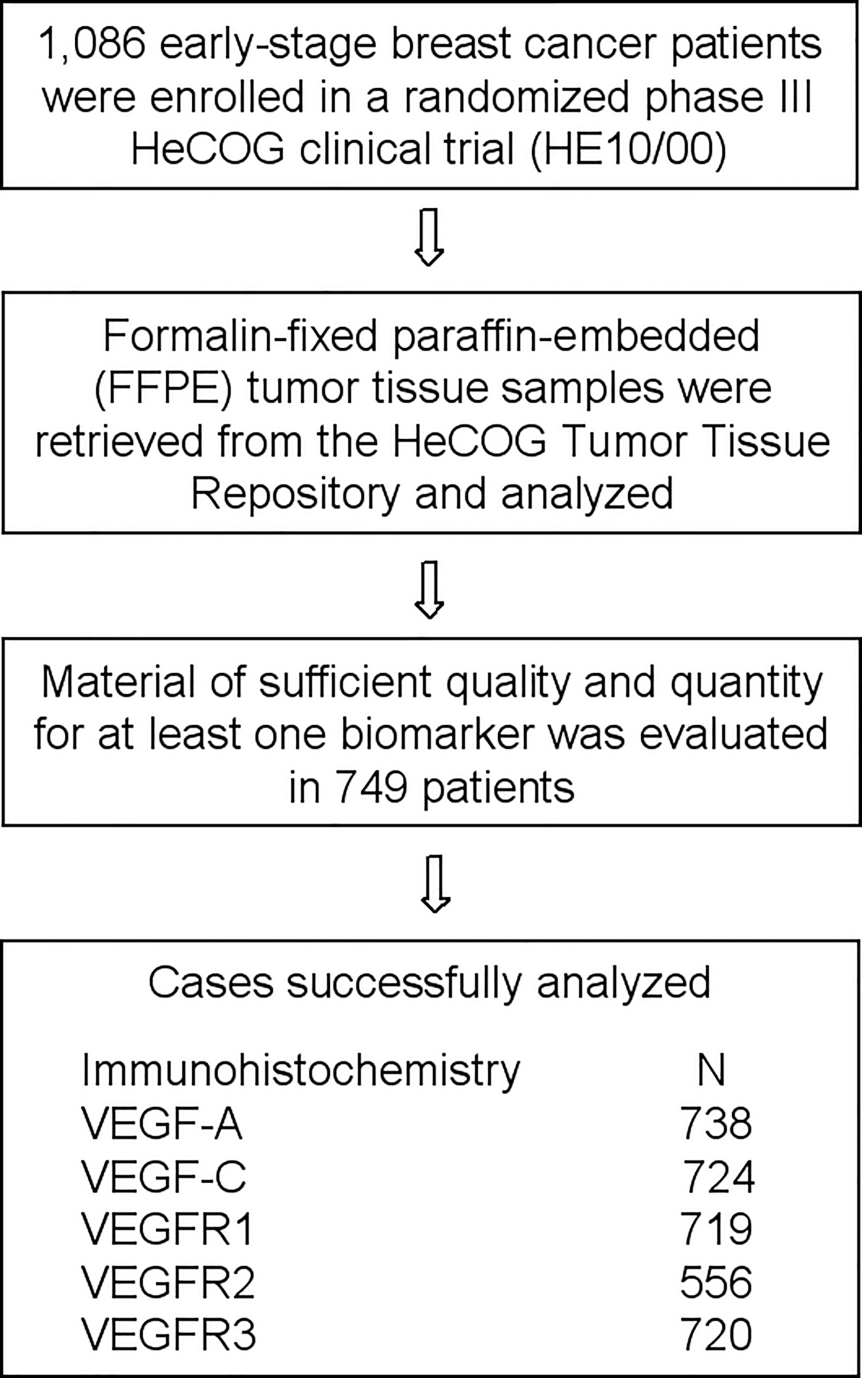
REMARK diagram detailing FFPE tumor tissue sample availability for the determination of immunohistochemical protein expression.

Formalin-fixed paraffin embedded (FFPE) tumor tissue samples were retrieved from the HeCOG Tumor Tissue Repository and a hematoxylin-eosin evaluation round was employed for obtaining cores for tissue microarrays (TMAs) construction. In 749 out of 1,086 patients the tumor tissue was adequate for the construction of TMAs containing 2 cores for each tumor, as well as positive and negative controls for the tested antibodies. Cases not represented or inadequate on the TMAs sections were re-cut from the original blocks and whole tissue sections were used for immunohistochemical analysis. The REMARK diagram is shown in **Fig 1.**

### Immunohistochemical procedure and interpretation

Immunohistochemistry was performed on serial 2.5 μm thick tissue sections from the TMAs or the original blocks. The histological sections were prepared at the Laboratory of Molecular Oncology of the Hellenic Foundation of Cancer Research/Aristotle University of Thessaloniki. The immunohistochemical procedure was performed using a Bond Max^TM^ autostainer machine (Leica Microsystems, Wetzlar, Germany). The primary antibodies used, their source, dilutions and staining conditions are presented in **Table 1**, as previously described[21].

**Table 1:** Primary antibodies, clone, source, dilution and staining conditions used in the present study.

Antibody	Clone/ Source	Dilution	Antigen Retrieval	Incubation Time
VEGF-A (m)	VG1 (1)	1:75	20΄/EDTA	60΄
VEGF-C (r, PL)	Z-CVC7 (2)	1:250	20΄/CA	Overnight
VEGFR1 (r)	RB-1527 (3)	1:450	15΄/CA	Overnight
VEGFR2 (r)	55B11 (4)	1:450	20΄/EDTA	Overnight
VEGFR3 (m)	KLT9 (5)	1:50	15΄/CA	Overnight

m, mouse; r, rabbit; PL, polyclonal; EDTA, ethylene diamine tetraacetate, PH 8.8; CA, citric acid, PH 6.0

(1), Dako, Glostrup, Denmark

(2), Zymed^TM^, Invitrogen, Carlsbad, CA

(3), Thermo Fisher Scientific, Fremont, CA

(4), Cell Signalling Technology, Beverly, MA

(5), Novocastra^TM^, Leica Biosystems, Newcastle Upon Tyne, UK.

All tissue sections were evaluated independently by two pathologists blinded to the patients’ clinicopathological data. The immunostaining was estimated only in areas with well-preserved tumor morphology and the assessment examined for the expression of the angiogenesis-related proteins in neoplastic cells. Positive tumoral stromal cells, inflammatory cells or endothelial cells were used as internal positive controls. Different scoring methods of tumor staining for VEGF-A, VEGF-C, VEGFR1, VEGFR2 and VEGFR3 were assessed: a) the percentage of stained neoplastic cells, irrespective of staining intensity, b) the H-score (range 0–300) and the Allred score (sum 0–8), taking account the percentage of stained neoplastic cells and the staining intensity. For each of the above biomarkers, cut-offs determining high and low protein expression groups were calculated through: a) ROC curves enquiring for the “optimal” cut-off value of each biomarker to predict the probability of patients having 5-year disease-free survival (DFS), b) median for % intensity, 50 for H-score (0–50 negative, 51–300 positive), 2 for Allred score (0–2 negative, 3–8 positive), c) distribution quartiles. We evaluated the prognostic significance of the scoring methods described above and selected the percentage of tumor stained cells with cut-offs determined using the 5-year DFS ROC curves.

ER, PgR, HER2 and Ki67 protein expression was centrally evaluated for the characterization of immunophenotypical breast cancer sybtypes. The staining procedures for estrogen receptor (ER, clone 6F11, dilution 1:70, NovocastraTM, Leica Biosystems, Newcastle, U.K), progesterone receptor (PgR, clone 1A6, dilution 1:70, NovocastraTM, Leica Biosystems), HER2 (A0485 polyclonal antibody, dilution 1:200, Dako, Glostrup, Denmark) and Ki67 (clone MIB-1, dilution 1:70, Dako) were performed using a Bond MaxTM autostainer (Leica Microsystems), as previously described in detail(25). Fluorescence in situ hybridization (FISH) analysis, using the ZytoLight® SPEC HER2/TOP2A/CEP17 triple-color probe (Z-2073, ZytoVision, Bremerhaven, Germany) was performed in all HER2 immunohistochemistry 2+ cases [25].

The evaluation of all sections was done by two experienced breast cancer pathologists, blinded as to the patients’ clinical characteristics and survival data. Briefly, HER2 protein expression was scored in a scale from 0 to 3+, the latter corresponding to uniform, intense membrane staining in >30% invasive tumor cells[26]; ER and PgR were considered positive if staining was present in ≥1% of tumor cell nuclei[27]; and, for Ki67, the expression was defined as low (<20%) or high (≥20%) based on the percentage of stained/unstained nuclei from the tumor areas[28].

### Statistical analysis

For the description of continuous variables the mean (standard deviation), median and range (min-max) were used, while categorical variables were presented as frequencies (%). The chi-square test was used for group comparisons of categorical data.

Statistical analyses focused on the examination of the aforementioned immunohistochemical markers with overall survival (OS) and DFS after adjusting for certain clinicopathological characteristics. Overall survival (OS) was defined as the time from the date of diagnosis with breast cancer to the date of patient’s death or last contact, while DFS was defined as the time from the date of diagnosis to documented first relapse, death without prior documented relapse or last contact, whichever occurred first. Surviving patients were censored at the date of last contact. Women who died without prior relapse were treated as events, that is as having had relapsed at the date of their death. Survival curves were estimated using the Kaplan-Meier method and compared across groups with the log-rank test. The associations between the examined factors and relapse/mortality rates were evaluated with hazard ratios (HRs) estimated with the Cox proportional hazards model. The proportional hazards assumption was tested by evaluating the statistical significance of the time-dependent association between each variable and relapse/mortality rates. The following parameters were studied in relation to DFS/OS: 1) clinicopathological, such as age (>median, ≤median), number of positive lymph nodes (0–3, ≥4), tumor size (≤2, >2cm), type of operation (breast-conserving surgery, modified radical mastectomy), breast cancer subtypes (luminal A, luminal B, luminal-HER2, HER2-enriched, triple-negative) 2) angiogenesis-related proteins (high vs. low expression), the cut-off values resulting from the ROC curve analyses (see methods above) hereafter referred to as 5-years DFS ROC curve cut-off. In multivariate analysis, we estimated the effect (HR) of each immunohistochemical marker adjusted for the effect of the clinicopathological parameters that were statistically significant (p<0.05) in the univariate analysis.

The above-indicated analyses were performed in the entire cohort of patients with available data for each marker examined and in the patient subgroups defined by: breast cancer subtypes (luminal A, luminal B, luminal-HER2, HER2-enriched, triple-negative), ER/PgR status (ER/PgR-positive, ER/PgR-negative) and HER2 status (HER2-positive, HER2-negative).

The statistical analyses were performed using the SAS software (SAS for Windows, version 9.3, SAS Institute Inc., Cary, NC). Statistical significance was set at 2-sided p = 0.05.

## Results

### Patient and tumor characteristics

Selected patient and tumor characteristics are shown in **Table 2** for the entire cohort and by treatment arm (these characteristics are also tabulated according to expression of one or more of the VEGF markers in **S3 Table**). The median age was 53.7 years (range 22–79). Most patients were postmenopausal (55.8%) and had invasive ductal carcinomas not otherwise specified (NOS, 81.8%), tumor size >2 cm (69.6%), positive lymph nodes ≥4 (52.0%), ER/PgR-positive (77.7%) and HER2-negative (76.9%) status. According to ER, PgR, HER2 and Ki67 status, tumors were classified as luminal A (ER- and/or PgR-positive, HER2-negative and Ki67 <20%; 40.0%), luminal B (ER- and/or PgR-positive, HER2-negative and Ki67 ≥20%; 22.8%), luminal-HER2 (ER- and/or PgR-positive and HER2-positive; 14.5%), HER2-enriched (ER-negative, PgR-negative, HER2-positive; 9.1%) and triple-negative breast cancer (TNBC, ER/PgR/HER2-negative; 13.6%).

**Table 2:** Basic patient and tumor characteristics in the entire cohort and by treatment arm (E-T-CMF, ET-CMF).

Parameter	Entire cohort (N = 749)	E-T-CMF (N = 373)	ET-CMF (N = 376)
	N (%)		
**Age**			
Mean (standard deviation)	53.5 (11.1)	53.2 (11.1)	53.9 (11.1)
Range	22–79	29–79	22–76
**Menopausal status**			
Postmenopausal	418 (55.8)	201 (53.9)	217 (57.7)
Premenopausal	325 (43.4)	171 (45.8)	154 (41.0)
Not reported	6 (0.8)	1 (0.3)	5 (1.3)
**Type of surgery**			
Modified radical mastectomy	482 (64.4)	246 (66)	236 (62.8)
Partial/Simple mastectomy	259 (34.6)	124 (33.2)	135 (36.0)
Not reported	8 (1.0)	3 (0.8)	5 (1.4)
**Tumor size**			
≤2	222 (29.6)	107 (28.7)	115 (30.6)
>2	521 (69.6)	265 (71.0)	256 (68.1)
Not reported	6 (0.8)	1 (0.3)	5 (1.3)
**Number of positive nodes**			
0–3	354 (47.2)	170 (45.6)	184 (49.0)
≥4	389 (52.0)	202 (54.1)	187 (49.7)
Not reported	6 (0.8)	1 (0.3)	5 (1.3)
**Histological classification**			
Invasive ductal	612 (81.8)	309 (82.8)	303 (80.6)
Invasive lobular	72 (9.6)	29 (7.8)	43 (11.5)
Mixed	47 (6.2)	27 (7.2)	20 (5.3)
Other	12 (1.6)	7 (1.9)	5 (1.3)
Not reported	6 (0.8)	1 (0.3)	5 (1.3)
**ER/PgR status**			
Informative*	736 (98.3)	364 (97.6)	372 (98.9)
Positive (ER and/or PgR positive)	572 (77.7)	280 (76.9)	292 (78.5)
Negative (ER and PgR negative)	164 (22.3)	84 (23.1)	80 (21.5)
**HER2 status**			
Informative**	745 (99.5)	372 (9.7)	373 (99.2)
Positive	172 (23.1)	89 (23.9)	83 (22.3)
Negative	573 (76.9)	283 (76.1)	290 (77.7)
**Subtypes**			
Informative***	718 (95.9)	356 (95.4)	362 (96.3)
Luminal A	287 (40.0)	139 (39.0)	148 (40.9)
Luminal B	164 (22.8)	82 (23.0)	82 (22.7)
Luminal-HER2	104 (14.5)	51 (14.3)	53 (14.6)
HER2-enriched	65 (9.1)	36 (10.1)	29 (8.0)
Triple-negative	98 (13.6)	48 (13.5)	50 (13.8)
**Histological grade**			
I-II	371 (49.6)	182 (48.8)	189 (50.2)
III-IV	368 (49.2)	189 (50.6)	179 (47.6)
Not reported	10 (1.4)	2 (0.6)	8 (2.2)
**Hormonal therapy**			
Yes	543 (72.4)	263 (70.5)	280 (74.5)
No	182 (24.2)	100 (26.8)	82 (21.8)
Unknown	24 (3.2)	10 (2.7)	14 (3.7)
**Radiotherapy**			
Yes	546 (72.8)	261 (70.0)	285 (75.8)
No	171 (22.8)	98 (26.3)	73 (19.4)
Unknown	32 (4.2)	14 (3.7)	18 (4.8)

*Data available from central evaluation of ER and PgR protein expression

**Data available from central evaluation of HER2 protein expression and HER2 amplification in 2+ cases

***Data available from central evaluation of ER/PgR and HER2 status and Ki67 protein expression.

### Associations among the angiogenic factors

The basic descriptive statistics for each of the angiogenic factors protein expression are shown in **S1 Table**. The cut-off values, identified through ROC analyses and used to dichotomize these markers according to their high/low expression, are also shown in this Table. The median percentage of stained tumor cells were 2.5%, 90.0%, 8.5%, 70.0% and 47.5% for VEGF-A, VEGF-C, VEGFR1, VEGFR2 and VEGFR3, respectively. High protein expression was observed in 11.8% (N = 87; cut-off = 55.0% VEGF-A positive cells), 80.8% (N = 585; cut-off = 72.5% VEGF-C positive cells), 28.1% (N = 202; cut-off = 43.5% VEGFR1 positive cells), 64.6% (N = 359; cut-off = 55.0% VEGFR2 positive cells) and 71.8% (N = 517; cut-off = 13.5% VEGFR3 positive cells) of the cases, respectively. The associations among these proteins are presented in **S2 Table**. Significant associations were observed among almost all proteins (all p-values ≤0.05), with the exception of the one between VEGF-C and VEGFR1 (chi-square test, p = 0.15).

### Associations between the angiogenic factors and clinicopathological parameters

The associations between the angiogenic factors and clinicopathological characteristics are presented in **S3 Table**. As is evident in this Table, some comparisons were carried out in the presence of very small cell counts, and therefore should be interpreted with care. Tumors with high VEGF-A protein expression, as compared to tumors with low expression were more frequently ER/PgR-negative (33.3% vs. 20.8%, chi-square test, p = 0.009) and HER2-positive (44.8% vs. 20.6%, p<0.001). In addition, tumors with high VEGFR1 expression, as compared to tumors with low expression, were more frequently HER2-positive (32.8% vs. 19.6%, p<0.001). Finally, tumors with high VEGFR3 expression were more frequently ER/PgR-negative (24.9% vs. 17.0%, p = 0.024) and HER2-positive (26.9% vs. 14.8%, p = 0.001).

Regarding the associations of the angiogenic factors with breast cancer subtypes, it was demonstrated that tumors with high VEGF-A expression, as compared to tumors with low expression, were more frequently of the HER2-positive subtypes (overall chi-square p<0.001). Moreover, tumors with high VEGFR1 expression were more frequently of the HER2-enriched subtype (p<0.001).

### Effect of angiogenic factors on outcome

Latest available survival status of the patients was retrieved from the HeCOG’s electronic database on April 2018. After a median follow-up period of 123.8 months (range 0.5–188.3), 257 DFS events and 201 deaths were recorded. Median OS was 172.5 (95% CI 172.5-not reached), while median DFS was not reached up to the date of the analyses.

The age-adjusted and multivariable-adjusted Cox regression analyses, for the entire cohort and for subgroups defined by breast cancer subtypes, ER/PgR status and HER2 status are presented in **Tables 3** and **4** for DFS and OS, respectively. In multivariable analyses, HRs were adjusted for breast surgery, tumor size, number of positive lymph nodes and breast cancer subtypes/subgroups (where appropriate). The number of patients/events in some of these subgroups is small and therefore results from these analyses should be interpreted with care.

**Table 3:** Hazard ratios and 95% CIs estimated from age-adjusted and multivariable-adjusted* Cox regression analyses with respect to DFS for the high vs. low expression of each of the angiogenesis-related proteins in the entire cohort and in selected subgroups.

	N of patients	N of events	Age-adjusted			Multivariable-adjusted*		
	High vs. low	High vs. low	HR	95% CI	p-value	HR	95% CI	p-value
**Entire Cohort**								
VEGF-A	87 vs. 651	20 vs. 234	0.60	0.38–0.95	**0.030**	0.57	0.36–0.92	**0.020**
VEGF-C	585 vs. 139	193 vs. 59	0.72	0.54–0.97	**0.028**	0.71	0.52–0.96	**0.025**
VEGFR1	202 vs. 517	61 vs. 187	0.82	0.61–1.09	0.174	0.80	0.59–1.09	0.151
VEGFR2	359 vs. 197	119 vs. 75	0.82	0.61–1.09	0.170	0.83	0.61–1.12	0.217
VEGFR3	517 vs. 203	183 vs. 63	1.20	0.90–1.60	0.209	1.29	0.95–1.74	0.102
**Luminal A**								
VEGF-A	22 vs. 258	6 vs. 82	0.91	0.40–2.08	0.815	1.17	0.50–2.71	0.716
VEGF-C	216 vs. 60	64 vs. 21	0.86	0.53–1.42	0.567	0.85	0.52–1.39	0.519
VEGFR1	74 vs. 202	18 vs. 70	0.64	0.38–1.08	0.094	0.69	0.41–1.15	0.156
VEGFR2	143 vs. 75	39 vs. 26	0.75	0.46–1.24	0.267	0.71	0.43–1.18	0.182
VEGFR3	185 vs. 89	59 vs. 27	1.20	0.76–1.90	0.426	1.28	0.81–2.03	0.297
**Luminal B**								
VEGF-A	14 vs. 149	5 vs. 51	0.99	0.40–2.49	0.990	1.53	0.58–3.98	0.388
VEGF-C	132 vs. 31	41 vs. 16	0.52	0.29–0.93	**0.028**	0.53	0.30–0.95	**0.034**
VEGFR1	44 vs. 114	11 vs. 42	0.63	0.32–1.22	0.171	0.57	0.29–1.12	0.103
VEGFR2	80 vs. 38	24 vs. 17	0.59	0.32–1.10	0.098	0.66	0.35–1.24	0.192
VEGFR3	111 vs. 48	41 vs. 12	1.34	0.69–2.60	0.382	1.65	0.86–3.17	0.130
**Luminal-HER2**								
VEGF-A	21 vs. 83	3 vs. 36	0.27	0.08–0.89	**0.031**	0.27	0.08–0.90	**0.034**
VEGF-C	86 vs. 17	35 vs. 4	1.87	0.66–5.30	0.240	1.67	0.59–4.76	0.335
VEGFR1	30 vs. 72	10 vs. 28	0.85	0.41–1.75	0.656	0.74	0.35–1.54	0.420
VEGFR2	48 vs. 25	19 vs. 11	0.86	0.41–1.83	0.703	0.97	0.46–2.07	0.941
VEGFR3	80 vs. 22	32 vs. 6	1.67	0.70–4.02	0.251	1.77	0.73–4.29	0.206
**HER-enriched**								
VEGF-A	18 vs. 47	3 vs. 21	0.34	0.10–1.15	0.083	0.44	0.13–1.53	0.198
VEGF-C	57 vs. 7	20 vs. 4	0.53	0.18–1.57	0.254	0.57	0.19–1.73	0.319
VEGFR1	36 vs. 27	11 vs. 13	0.60	0.27–1.35	0.215	0.70	0.31–1.58	0.387
VEGFR2	30 vs. 17	11 vs. 8	0.74	0.29–1.88	0.526	0.87	0.33–2.26	0.771
VEGFR3	56 vs. 8	19 vs. 4	0.57	0.19–1.68	0.305	0.69	0.23–2.04	0.504
**TNBC**								
VEGF-A	11 vs. 85	3 vs. 34	0.66	0.20–2.21	0.503	0.52	0.16–1.74	0.291
VEGF-C	74 vs. 19	25 vs. 11	0.43	0.21–0.88	**0.020**	0.44	0.21–0.91	**0.027**
VEGFR1	13 vs. 82	9 vs. 27	2.92	1.36–6.27	**0.006**	2.74	1.26–5.98	**0.011**
VEGFR2	49 vs. 33	22 vs. 10	1.44	0.68–3.08	0.344	1.35	0.63–2.88	0.443
VEGFR3	70 vs. 26	28 vs. 9	1.25	0.59–2.67	0.566	1.05	0.49–2.28	0.899
**ER/PgR-positive**								
VEGF-A	58 vs. 505	14 vs. 173	0.67	0.39–1.15	0.143	0.71	0.40–1.25	0.236
VEGF-C	444 vs. 112	143 vs. 43	0.81	0.58–1.15	0.239	0.80	0.57–1.14	0.223
VEGFR1	151 vs. 398	40 vs. 142	0.70	0.49–0.99	**0.046**	0.69	0.48–0.98	**0.041**
VEGFR2	276 vs. 141	84 vs. 54	0.74	0.52–1.04	0.081	0.76	0.53–1.07	0.115
VEGFR3	383 vs. 166	133 vs. 47	1.32	0.95–1.85	0.099	1.43	1.01–2.01	**0.042**
**ER/PgR-negative**								
VEGF-A	29 vs. 133	6 vs. 55	0.47	0.20–1.09	0.078	0.47	0.20–1.11	0.086
VEGF-C	131 vs. 26	45 vs. 15	0.46	0.26–0.84	**0.011**	0.46	0.25–0.85	**0.013**
VEGFR1	49 vs. 110	20 vs. 40	1.23	0.72–2.12	0.444	1.32	0.70–2.47	0.391
VEGFR2	79 vs. 50	33 vs. 18	1.09	0.61–1.95	0.762	1.06	0.59–1.89	0.842
VEGFR3	127 vs. 34	47 vs. 13	1.00	0.54–1.86	0.993	0.93	0.50–1.75	0.827
**HER2-positive**								
VEGF-A	39 vs. 133	6 vs. 58	0.30	0.13–0.71	**0.006**	0.32	0.14–0.74	**0.008**
VEGF-C	145 vs. 24	56 vs. 8	1.20	0.57–2.52	0.639	1.12	0.53–2.37	0.773
VEGFR1	66 vs. 101	21 vs. 42	0.74	0.44–1.26	0.270	0.71	0.41–1.23	0.224
VEGFR2	80 vs. 42	31 vs. 19	0.81	0.46–1.45	0.486	0.91	0.51–1.64	0.765
VEGFR3	138 vs. 30	52 vs. 10	1.20	0.60–2.36	0.608	1.17	0.59–2.33	0.648
**HER2-negative**								
VEGF-A	48 vs. 514	14 vs. 174	0.84	0.49–1.45	0.528	0.89	0.51–1.55	0.680
VEGF-C	437 vs. 115	135 vs. 51	0.62	0.45–0.86	**0.004**	0.64	0.46–0.89	**0.008**
VEGFR1	135 vs. 413	40 vs. 143	0.82	0.58–1.17	0.269	0.85	0.59–1.23	0.396
VEGFR2	278 vs. 153	88 vs. 54	0.83	0.59–1.16	0.269	0.80	0.56–1.13	0.197
VEGFR3	375 vs. 173	129 vs. 53	1.18	0.86–1.63	0.305	1.32	0.94–1.84	0.108

*Adjusted for breast surgery, tumor size, number of positive lymph nodes and breast cancer subtypes (where appropriate).

N, number; HR, hazard ratio; CI, confidence interval; TNBC, triple-negative breast cancer.

Significant p-values are shown in bold.

**Table 4:** Hazard ratios and 95% CIs estimated from age-adjusted and multivariable-adjusted* Cox regression analyses with respect to OS for the high vs. low expression of each of the angiogenesis-related proteins in the entire cohort and in selected subgroups.

	N of patients	N of events	Age-adjusted			Multivariable-adjusted*		
	High vs. low	High vs. low	HR	95% CI	p-value	HR	95% CI	p-value
**Entire Cohort**								
VEGF-A	87 vs. 651	19 vs. 180	0.79	0.49–1.26	0.316	0.77	0.48–1.26	0.301
VEGF-C	585 vs. 139	148 vs. 49	0.68	0.49–0.94	**0.020**	0.64	0.46–0.89	**0.008**
VEGFR1	202 vs. 517	46 vs. 147	0.81	0.58–1.14	0.227	0.80	0.57–1.14	0.221
VEGFR2	359 vs. 197	90 vs. 58	0.82	0.59–1.15	0.255	0.81	0.58–1.14	0.236
VEGFR3	517 vs. 203	142 vs. 49	1.22	0.88–1.70	0.225	1.29	0.92–1.81	0.142
**Luminal A**								
VEGF-A	22 vs. 258	5 vs. 53	1.23	0.49–3.09	0.656	1.72	0.68–4.38	0.253
VEGF-C	216 vs. 60	41 vs. 15	0.82	0.45–1.49	0.518	0.79	0.44–1.44	0.442
VEGFR1	74 vs. 202	12 vs. 46	0.69	0.36–1.30	0.247	0.76	0.40–1.43	0.392
VEGFR2	143 vs. 75	23 vs. 18	0.67	0.36–1.24	0.202	0.61	0.32–1.15	0.128
VEGFR3	185 vs. 89	38 vs. 18	1.21	0.69–2.13	0.510	1.25	0.71–2.22	0.436
**Luminal B**								
VEGF-A	14 vs. 149	5 vs. 46	1.19	0.47–3.00	0.719	1.85	0.70–4.88	0.217
VEGF-C	132 vs. 31	36 vs. 15	0.54	0.29–0.98	**0.044**	0.53	0.29–0.98	**0.043**
VEGFR1	44 vs. 114	10 vs. 38	0.66	0.33–1.32	0.235	0.64	0.31–1.29	0.212
VEGFR2	80 vs. 38	20 vs. 17	0.48	0.25–0.91	**0.026**	0.56	0.29–1.09	0.090
VEGFR3	111 vs. 48	37 vs. 11	1.41	0.70–2.83	0.334	1.68	0.85–3.32	0.135
**Luminal-HER2**								
VEGF-A	21 vs. 83	3 vs. 29	0.38	0.12–1.26	0.115	0.36	0.11–1.21	0.098
VEGF-C	86 vs. 17	29 vs. 3	2.32	0.70–7.66	0.167	2.18	0.65–7.25	0.205
VEGFR1	30 vs. 72	8 vs. 23	0.91	0.41–2.05	0.825	0.82	0.36–1.86	0.639
VEGFR2	48 vs. 25	17 vs. 6	1.57	0.62–4.00	0.343	1.72	0.66–4.44	0.265
VEGFR3	80 vs. 22	26 vs. 5	1.69	0.65–4.44	0.283	1.94	0.73–5.16	0.184
**HER-enriched**								
VEGF-A	18 vs. 47	3 vs. 15	0.59	0.17–2.06	0.404	1.04	0.29–3.82	0.948
VEGF-C	57 vs. 7	14 vs. 4	0.28	0.09–0.89	**0.031**	0.27	0.08–0.89	**0.032**
VEGFR1	36 vs. 27	7 vs. 11	0.52	0.20–1.38	0.192	0.61	0.23–1.61	0.316
VEGFR2	30 vs. 17	8 vs. 6	0.94	0.30–2.89	0.912	0.99	0.31–3.19	0.991
VEGFR3	56 vs. 8	13 vs. 4	0.58	0.16–2.06	0.400	0.67	0.19–2.37	0.538
**TNBC**								
VEGF-A	11 vs. 85	3 vs. 30	0.65	0.19–2.19	0.490	0.61	0.18–2.06	0.428
VEGF-C	74 vs. 19	22 vs. 10	0.42	0.20–0.90	**0.025**	0.42	0.19–0.90	**0.026**
VEGFR1	13 vs. 82	7 vs. 25	1.95	0.84–4.55	0.123	1.80	0.76–4.28	0.184
VEGFR2	49 vs. 33	19 vs. 10	1.14	0.53–2.47	0.740	1.10	0.50–2.39	0.815
VEGFR3	70 vs. 26	25 vs. 8	1.28	0.57–2.87	0.550	1.06	0.47–2.40	0.895
**ER/PgR-positive**								
VEGF-A	58 vs. 505	13 vs. 131	0.86	0.49–1.53	0.617	0.98	0.54–1.77	0.934
VEGF-C	444 vs. 112	109 vs. 34	0.83	0.56–1.22	0.349	0.77	0.52–1.13	0.182
VEGFR1	151 vs. 398	31 vs. 108	0.75	0.50–1.12	0.159	0.74	0.49–1.12	0.155
VEGFR2	276 vs. 141	61 vs. 41	0.73	0.49–1.08	0.118	0.77	0.51–1.15	0.202
VEGFR3	383 vs. 166	102 vs. 35	1.40	0.95–2.07	0.086	1.49	1.00–2.21	0.048
**ER/PgR-negative**								
VEGF-A	29 vs. 133	6 vs. 45	0.60	0.25–1.42	0.243	0.67	0.28–1.62	0.376
VEGF-C	131 vs. 26	36 vs. 14	0.37	0.20–0.69	**0.002**	0.37	0.20–0.71	**0.003**
VEGFR1	49 vs. 110	14 vs. 36	0.94	0.51–1.76	0.852	1.07	0.53–2.15	0.854
VEGFR2	79 vs. 50	27 vs. 16	1.07	0.57–2.02	0.836	1.01	0.53–1.91	0.977
VEGFR3	127 vs. 34	38 vs. 12	0.97	0.49–1.90	0.921	0.92	0.46–1.84	0.820
**HER2-positive**								
VEGF-A	39 vs. 133	6 vs. 45	0.46	0.20–1.09	0.079	0.48	0.20–1.13	0.094
VEGF-C	145 vs. 24	44 vs. 7	1.06	0.47–2.35	0.895	0.97	0.43–2.18	0.935
VEGFR1	66 vs. 101	15 vs. 35	0.71	0.39–1.31	0.275	0.71	0.38–1.33	0.286
VEGFR2	80 vs. 42	26 vs. 12	1.21	0.60–2.41	0.593	1.29	0.63–2.62	0.481
VEGFR3	138 vs. 30	40 vs. 9	1.11	0.53–2.29	0.787	1.18	0.56–2.49	0.658
**HER2-negative**								
VEGF-A	48 vs. 514	13 vs. 134	1.02	0.58–1.81	0.935	1.07	0.60–1.92	0.811
VEGF-C	437 vs. 115	103 vs. 42	0.61	0.43–0.88	**0.008**	0.59	0.41–0.86	**0.005**
VEGFR1	135 vs. 413	31 vs. 111	0.84	0.56–1.25	0.378	0.85	0.56–1.29	0.449
VEGFR2	278 vs. 153	64 vs. 45	0.73	0.50–1.07	0.108	0.70	0.47–1.03	0.069
VEGFR3	375 vs. 173	101 vs. 40	1.27	0.87–1.83	0.214	1.31	0.90–1.92	0.162

*Adjusted for breast surgery, tumor size, number of positive lymph nodes and breast cancer subtypes (where appropriate).

N, number; HR, hazard ratio; CI, confidence interval; TNBC, triple-negative breast cancer.

Significant p-values are shown in bold.

### Disease-free survival

The direction and statistical significance of the estimated HRs were the same in both age-adjusted and multivariate analyses. Among all patients, high VEGF-A and VEGF-C protein expression was associated with increased DFS (**Table 3**). When patients were analyzed according to breast cancer subtypes, high as compared to low expression of VEGF-A was a favorable factor for DFS among luminal-HER2 and HER2-positive patients. High VEGF-C expression was associated with increased DFS in patients with luminal B, TNBC, ER/PgR-negative and HER2-negative tumors. In the ER/PgR-positive subgroup, high VEGFR3 expression was associated with increased relapse (HR = 1.43, 95% CI 1.01–2.01, Wald’s p = 0.042). High vs. low expression of VEGFR1 protein was deemed unfavorable for the risk of relapse in the TNBC subgroup (HR = 2.74, 95% CI 1.26–5.98, p = 0.011), whereas the opposite was observed among the ER/PgR positive breast cancer patients (HR = 0.69, 95% CI 0.48–0.98, p = 0.041). Of note the association of high VEGFR1 expression with DFS was not statistically significant in the entire population of women (HR = 0.80, 95% CI 0.59–1.09, p = 0.151). None of the examined factors was statistically significant among women with HER-enriched or luminal A tumors.

### Overall survival

**Fig 2:**
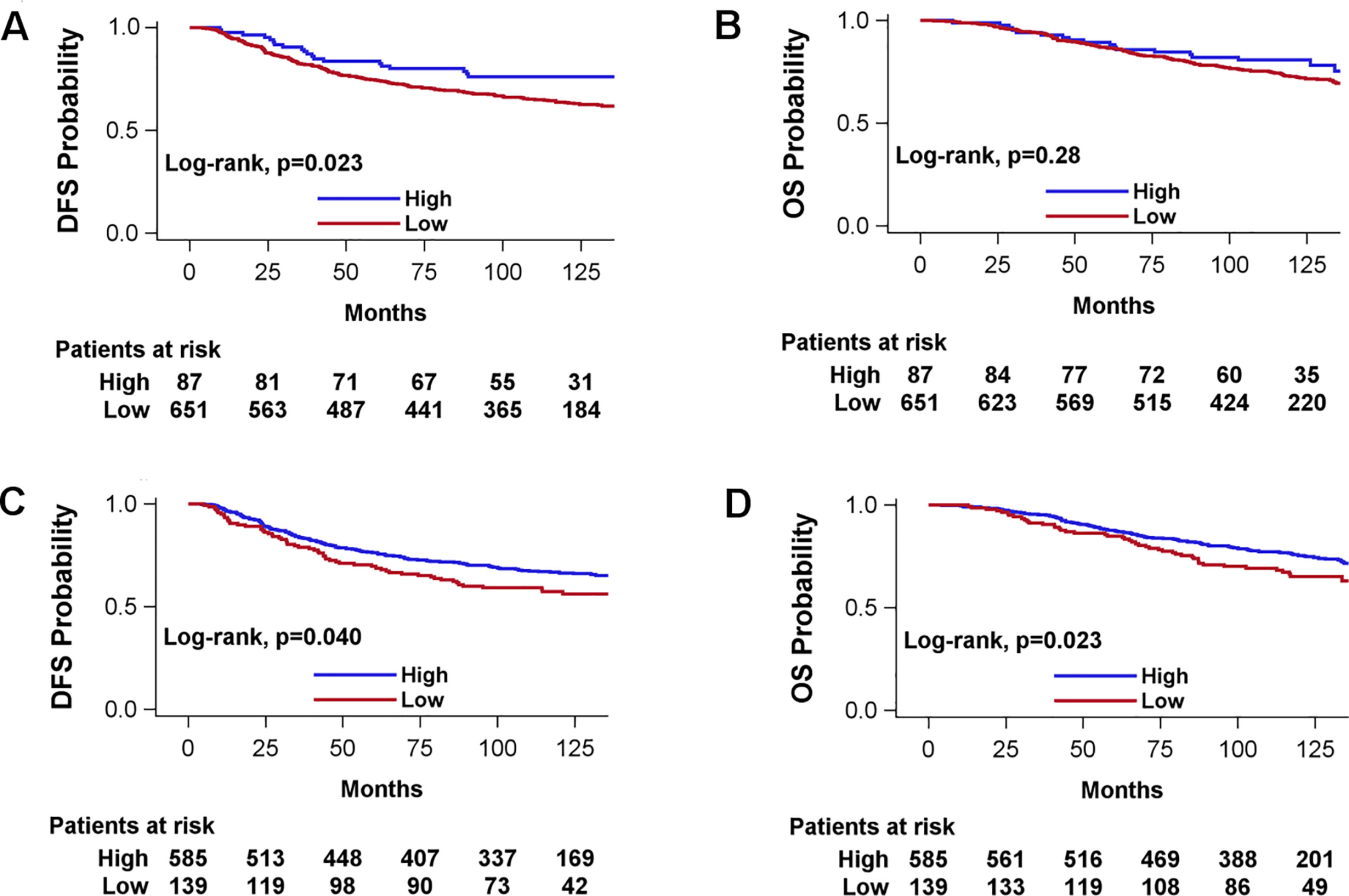
Kaplan-Meier curves according to VEGF-A (A, B) and VEGF-C (C, D) protein expression (5-years DFS ROC curve cut-off). A, C: DFS; B, D: OS.

In the entire population of women only high as compared to low VEGF-C protein expression was associated with increased survival (HR = 0.64, 95% CI 0.46–0.89, Wald’s p = 0.008) (**Table 4**). High expression of VEGF-C was also favorably associated with survival in patients with luminal B, HER-enriched and TNBC breast cancer subtypes and ER/PgR-negative and HER2-negative tumors. In addition, high vs. low expression of VEGFR2 was associated with prolonged survival among luminal B patients but only in the age-adjusted analysis. Among ER/PgR-positive patients VEGFR3 (high vs. low) was marginally statistically significantly associated with increased mortality (HR = 1.49, 95% CI 1.00–2.21, p = 0.048). None of the examined factors was statistically significantly associated with survival among women with HER2-positive, luminal-HER2 and luminal A breast cancer subtypes. Kaplan-Meier curves of DFS probability and OS probability according to VEGF-A and VEGF-C expression are presented in **Fig 2**.

Results from the univariate Cox regression analyses in the entire cohort for each of the clinicopathological parameters are presented in **S4 Table**. Breast conserving surgery, lower tumor size, number of positive lymph nodes and positive ER/PgR status were associated with improved outcome in terms of both DFS and OS.

## Discussion

Herein we analyzed the immunohistochemical expression of VEGF family members in a large cohort of early breast cancer patients treated in a randomized trial with long-term follow-up and evaluated their association with clinicopathological parameters, clinical subtypes and survival outcome.

Up-regulation of VEGF family members has been found in invasive breast carcinomas by immunohistochemical, PCR or Western blot approaches[1, 8–21]. However, in most series the analysis of the angiogenic markers has been investigated by immunohistochemistry and several studies have examined their prognostic and/or predictive value with some supporting[8, 14, 18, 22] and others refuting an adverse effect[10, 13, 15, 23, 29]. The conflicting results may be due to the molecular heterogeneity of the disease, to different antibodies used and to variability in the selected cut-offs.

In our study, conducted in the pre-trastuzumab era, we found that high VEGF-A, VEGF-C, VEGFR2 and VEGFR3 protein expression was associated with certain adverse prognostic factors, such as ER/PgR-negative and/or HER2-positive status, suggesting that high protein expression of VEGF family members might have a negative prognostic impact on DFS or OS. Our findings are consistent with the results of recent studies[19, 29, 30] and suggest that subgroups of patients with enhanced tumor angiogenesis seem to have an unfavorable profile, while signaling through VEGF receptors in cancer cells can promote events associated with tumor progression. Existing evidence suggests that HER2 activation is one of the several mechanisms that promote angiogenesis and that HER2-amplified breast cancers have increased angiogenesis[31]. Data from preclinical models report that in NIH 3T3 fibroblasts, which have been transfected to overexpress HER2, VEGF-A production was increased and this overproduction was blocked using a monoclonal antibody directed to HER2[32]. Therefore, the evaluation of VEGF family members could prove to be more useful when analyzed in combination with other markers, with potential to provide information regarding the profile of breast cancer aggressiveness.

In the analysis of angiogenic factors coexpression, an association between the ligands and receptors was observed. This observation suggests an “intracrine/autocrine” regulatory mechanism, supporting cancer cell autonomy, as has previously been suggested[18, 33] and supports the view of complementary functions of the angiogenesis pathways in the neoplastic cells.

The issue of whether angiogenesis is enhanced in distinct breast cancer subtypes has been discussed in the literature[29, 30, 34–36]. Gene expression profiling has been used to classify breast carcinomas into molecular subtypes with significant differences in incidence, risk factors, prognosis and treatment sensitivity[37]. By using a panel of immunohistochemical markers, breast carcinomas have been classified into phenotypic subtypes, quite similar to those identified by gene expression profiling[25, 29, 34, 35, 38–40], although the correlation between gene expression and immunohistochemical subtyping has not been shown to be perfect[41]. Several studies have reported an enhanced angiogenesis, as assessed by VEGF-A, VEGF-A/VEGFR2, VEGF-C or VEGFR3 immunohistochemical or mRNA expression in luminal B, luminal-HER2, HER2-enriched and TNBC/basal-like tumors compared to the luminal A subtype[29, 30, 34, 35, 42]. In our study, tumors with high expression of VEGF-A and VEGFR1, compared with tumors with low expression, were more frequently of the HER2-positive subtypes (luminal-HER2, HER2-enriched), while tumors with high VEGFR3 expression were more frequently of the HER2-positive and TNBC subtypes. Breast cancer subtypes, defined by our classification scheme, have shown different prognosis, with luminal A tumors having better prognosis[25]. It seems that the expression of VEGF family members in breast cancer, when elevated, is associated with more aggressive cancer phenotypes and therefore more aggressive tumor biology.

According to our findings, the association of some angiogenic proteins with an aggressive tumor profile, namely the fact that higher expression was to be expected when the tumor was more aggressive, was not reflected by a negative prognostic effect on DFS or OS. Existing data regarding the prognostic significance of angiogenic markers, when studied by immunohistochemistry, are controversial[8, 10, 13–15, 18, 22, 23, 29]. Contrary to expectations, high VEGF-A expression, when analyzed in the entire cohort, was associated with favorable DFS, in both age-adjusted and multivariable-adjusted analyses. In the subgroup analyses, VEGF-A retained its favorable prognostic value for DFS in luminal-HER2 and HER2-positive tumors, however, this should be interpreted with caution, due to the small number of events in the high VEGF-A category. Furthermore, high VEGF-C expression, when analyzed in the entire cohort, was associated with favorable DFS and OS, in both age-adjusted and multivariable-adjusted analyses. Additionally, high VEGF-C expression predicted for better DFS and OS in HER2-negative patients and in patients of the luminal B and TNBC subtype, while high VEGFR1 expression was associated with favorable DFS in ER/PgR-positive patients and unfavorable DFS in patients of the TNBC subtype. Using a large number of breast cancer cases identified from the Nurses’ Health Study, Liu et al. found that the VEGF-A associated adverse effects on breast cancer specific mortality and distant recurrence was only observed in luminal A tumors and not in luminal B, HER2-positive and basal-like/triple-negative cancers(29). Interestingly, the authors reported a VEGF-A associated decreased risk of overall mortality in patients with basal-like tumors. In the study by Kourea et al, where no direct anti-VEGF treatment was administered, high immunohistochemical expression of VEGFR1 and co-expression of VEGFR1/VEGFR2 were associated with better survival, irrespectively of breast cancer subtyping[21]. Based on our observations, one could hypothesize that among ER/PgR-negative or TNBC tumors, subsets of less aggressive cancers with better prognosis may be identified, according to their angiogenic profile and suggest that more than one of the components of the VEGF signaling pathway rather than an individual member may affect prognosis of breast cancer patients. This issue should be addressed in larger cohorts, where the expression of individual VEGF ligands would be evaluated in conjunction with their respective receptors, which might eventually resolve the many discrepant results appearing in the literature and shed more light in the apparent interactions between the multiple players in the VEGF signaling pathway. For instance, when looking more carefully at the 9 TNBC patients with high VEGFR1 protein expression that was associated with significantly (HR = 2.74, 95% CI 1.26–5.98, p = 0.011) decreased DFS, 7 of the 9 TNBC patients were found to have low VEGF-A protein expression. It is unclear whether the low VEGF-A protein expression seen in most of these patients was responsible for the up-regulation of the VEGFR1 and the observed decreased DFS in these patients.

The contradiction among the original studies, as well as among some interesting recent meta-analyses[43–44], concerning the prognostic significance of most of the angiogenesis-related proteins has hindered their clinical utility in breast cancer patients. One has to remember however, that in most of the existing studies, including ours, the findings are not purely prognostic, since most if not all studies included some type of treatment (hormonal treatment or chemotherapy). In our trial, concurrent or dose-dense sequential epirubicin and paclitaxel were administered, followed by dose-dense CMF. It appears therefore that high expression of some VEGF proteins in early-stage breast cancer patients with aggressive tumors, could plausibly lead to better DFS and OS, when such patients had received in the adjuvant setting a very effective regimen, like the one administered in our study.

The present study has some limitations. High VEGF-A expression was observed in a rather small proportion of tumors (11.8%), therefore the results concerning its prognostic utility may not be conclusive. Limitations regarding immunohistochemistry, as a method for assessing angiogenesis-related proteins in FFPE samples, have been described[45–46]. In addition, caution is warranted when comparing the present data related with immunohistochemical subtyping with those from studies employing gene expression profiling for breast cancer subtyping; the two approaches do not perfectly fit. Moreover, our study was conducted in the pre-trastuzumab era, thus, it is unclear if the results related to associations of angiogenic markers with HER2-positive tumors are applicable to patients treated in the trastuzumab era.

In conclusion, high expression of angiogenesis-related proteins is associated with adverse clinicopathological parameters in early-stage breast cancer patients and may be surrogate markers of biologically distinct subgroups of ER/PgR-negative or TNBC tumors with superior outcome. The multiple associations identified among ligands and receptors highlight potential intracellular pathways in the tumor cells. Further studies are undoubtedly needed in order to validate our results in independent cohorts.

## Supporting information

S1 Table Descriptive statistics for the five angiogenesis-related proteins (calculated as the percentage of tumor stained cells). (PDF)

S2 Table Associations among the angiogenesis-related proteins (using 5-years ROC curve cut-offs). Data presented as N (%). p-values of the chi-square test are shown.
(PDF)

S3 Table Associations between the angiogenesis-related proteins (using 5-years ROC curve cut-offs) and selected clinicopathological parameters. Data presented as N (%). p-values of the chi-square test are shown.
(PDF)

S4 Table Hazard ratios (95% CIs) estimated from univariate Cox regression for selected clinicopathological parameters. (PDF)

S1 Dataset (XLSX)
