# Supplementary material for: Associations of angiogenesis-related proteins with specific prognostic factors, breast cancer subtypes and survival outcome in early-stage breast cancer patients. A Hellenic Cooperative Oncology Group (HeCOG) trial
Source: PLoS One. 2018 Jul 31;13(7):e0200302. doi: 10.1371/journal.pone.0200302 (PMC6067711; doi:10.1371/journal.pone.0200302)
Supplement: S1 Table — (PDF) [file pone.0200302.s001.pdf]

S1 Table. Descriptive statistics for the five angiogenesis-related proteins (calculated as the percentage of tumor stained cells).

	N	Mean	Std	Median	Min	Max	Cut-off for determining high vs. low expression
VEGF-A	738	16.1	23.3	2.5	0.0	92.5	55.0% of VEGF-A positive cells
VEGF-C	724	83.1	18.5	90.0	1.0	100.0	72.5% of VEGF-C positive cells
VEGFR1	719	25.6	30.5	8.5	0.0	95.0	43.5% of VEGFR1 positive cells
VEGFR2	556	59.3	30.1	70.0	0.0	100.0	55.0% of VEGFR2 positive cells
VEGFR3	720	44.0	31.8	47.5	0.0	95.0	13.5% of VEGFR3 positive cells

N, number; Std, standard deviation.
