# Supplementary material for: Associations of angiogenesis-related proteins with specific prognostic factors, breast cancer subtypes and survival outcome in early-stage breast cancer patients. A Hellenic Cooperative Oncology Group (HeCOG) trial
Source: PLoS One. 2018 Jul 31;13(7):e0200302. doi: 10.1371/journal.pone.0200302 (PMC6067711; doi:10.1371/journal.pone.0200302)
Supplement: S2 Table — Data presented as N (%). p-values of the chi-square test are shown. (PDF) [file pone.0200302.s002.pdf]

**S2 Table.** Associations among the angiogenesis-related proteins (using 5-years ROC curve cut-offs). Data presented as N (%). p-values of the chi-square test are shown.

| | | **VEGF-A** | | | **VEGF-C** | | | **VEGFR1** | | | **VEGFR2** | | | **VEGFR3** | | |
|---|---|---|---|---|---|---|---|---|---|---|---|---|---|---|---|---|
| | | **Low** | **High** | **p-value** | **Low** | **High** | **p-value** | **Low** | **High** | **p-value** | **Low** | **High** | **p-value** | **Low** | **High** | **p-value** |
| VEGF-A | High | 651 (100.0) | | - | 80 (13.8) | 7 (5.1) | **0.005** | 40 (20.0) | 46 (8.9) | **<0.001** | 55 (15.6) | 14 (7.1) | **0.004** | 83 (16.1) | 4 (2.0) | **<0.001** |
| | Low | | 87 (100.0) | | 500 (86.2) | 131 (94.9) | | 160 (80.0) | 469 (91.1) | | 298 (84.4) | 182 (92.9) | | 431 (83.9) | 199 (98.0) | |
| VEGF-C | High | 500 (79.2) | 80 (92.0) | **0.005** | 585 (100.0) | | - | 169 (84.1) | 400 (79.4) | 0.15 | 288 (82.8) | 141 (71.9) | **0.003** | 425 (84.0) | 146 (72.6) | **0.001** |
| | Low | 131 (20.8) | 7 (8.0) | | | 139 (100.0) | | 32 (15.9) | 104 (20.6) | | 60 (17.2) | 55 (28.1) | | 81 (16.0) | 55 (27.4) | |
| VEGFR1 | High | 160 (25.4) | 40 (46.5) | **<0.001** | 169 (29.7) | 32 (23.5) | 0.15 | 202 (100.0) | | - | 116 (33.5) | 36 (18.5) | **<0.001** | 169 (33.5) | 29 (14.6) | **<0.001** |
| | Low | 469 (74.6) | 46 (53.5) | | 400 (70.3) | 104 (76.5) | | | 517 (100.0) | | 230 (66.5) | 159 (81.5) | | 336 (66.5) | 169 (85.4) | |
| VEGFR2 | High | 298 (62.1) | 55 (79.7) | **0.004** | 288 (67.1) | 60 (52.2) | **0.003** | 116 (76.3) | 230 (59.1) | **<0.001** | 359 (100.0) | | - | 274 (68.8) | 73 (51.4) | **<0.001** |
| | Low | 182 (37.9) | 14 (20.3) | | 141 (32.9) | 55 (47.8) | | 36 (23.7) | 159 (40.9) | | | 197 (100.0) | | 124 (31.2) | 69 (48.6) | |
| VEGFR3 | High | 431 (68.4) | 83 (95.4) | **<0.001** | 425 (74.4) | 81 (59.6) | **0.001** | 169 (85.4) | 336 (66.5) | **<0.001** | 274 (79.0) | 124 (64.2) | **<0.001** | 517 (100.0) | | - |
| | Low | 199 (31.6) | 4 (4.6) | | 146 (25.6) | 55 (40.4) | | 29 (14.6) | 169 (33.5) | | 73 (21.0) | 69 (35.8) | | | 203 (100.0) | |

Significant p-values are shown in bold.
