# Supplementary material for: Associations of angiogenesis-related proteins with specific prognostic factors, breast cancer subtypes and survival outcome in early-stage breast cancer patients. A Hellenic Cooperative Oncology Group (HeCOG) trial
Source: PLoS One. 2018 Jul 31;13(7):e0200302. doi: 10.1371/journal.pone.0200302 (PMC6067711; doi:10.1371/journal.pone.0200302)
Supplement: S3 Table — Data presented as N (%). p-values of the chi-square test are shown. (PDF) [file pone.0200302.s003.pdf]

S3 Table. Associations between the angiogenesis-related proteins (using 5-years ROC curve cut-offs) and selected clinicopathological parameters. Data presented as N (%). p-values of the chi-square test are shown.

		VEGF-A			VEGF-C			VEGFR1			VEGFR2			VEGFR3		
		High	Low	p-value	High	Low	p-value	High	Low	p-value	High	Low	p-value	High	Low	p-value
**Age (median cut-off)**	<53.7	38 (44.2)	329 (50.9)	0.24	300 (51.7)	61 (44.2)	0.11	106 (52.7)	251 (49.0)	0.37	181 (50.7)	90 (46.4)	0.33	276 (53.8)	85 (42.3)	**0.006**
	≥53.7	48 (55.8)	317 (49.1)		280 (48.3)	77 (55.8)		95 (47.3)	261 (51.0)		176 (49.3)	104 (53.6)		237 (46.2)	116 (57.7)	
**Menopausal status**	Postmenopausal	52 (60.5)	360 (55.7)	0.41	317 (54.7)	86 (62.3)	0.10	102 (50.7)	300 (58.6)	0.057	198 (55.5)	117 (60.3)	0.27	264 (51.5)	134 (66.7)	**<0.001**
	Premenopausal	34 (39.5)	286 (44.3)		263 (45.3)	52 (37.7)		99 (49.3)	212 (41.4)		159 (44.5)	77 (39.7)		249 (48.5)	67 (33.3)	
**Histological grade**	I-II	38 (44.2)	326 (50.8)	0.25	293 (50.9)	60 (43.5)	0.12	98 (48.8)	256 (50.4)	0.69	175 (49.6)	94 (48.5)	0.80	255 (50.0)	97 (48.5)	0.72
	III-IV	48 (55.8)	316 (49.2)		283 (49.1)	78 (56.5)		103 (51.2)	252 (49.6)		178 (50.4)	100 (51.5)		255 (50.0)	103 (51.5)	
**Number of positive lymph nodes**	0-3	40 (46.5)	308 (47.7)	0.84	276 (47.6)	68 (49.3)	0.72	94 (46.8)	244 (47.7)	0.83	174 (48.7)	90 (46.4)	0.60	246 (48.0)	94 (46.8)	0.78
	≥4	46 (53.5)	338 (52.3)		304 (52.4)	70 (50.7)		107 (53.2)	268 (52.3)		183 (51.3)	104 (53.6)		267 (52.0)	107 (53.2)	
**Tumor size**	≤2	27 (31.4)	190 (29.4)	0.71	176 (30.3)	36 (26.1)	0.32	64 (31.8)	146 (28.5)	0.38	99 (27.7)	53 (27.3)	0.92	153 (29.8)	60 (29.9)	0.995
	>2	59 (68.6)	456 (70.6)		404 (69.7)	102 (73.9)		137 (68.2)	366 (71.5)		258 (72.3)	141 (72.7)		360 (70.2)	141 (70.1)	
**ER/PgR status**	Negative	29 (33.3)	133 (20.8)	**0.009**	131 (22.8)	26 (18.8)	0.32	49 (24.5)	110 (21.7)	0.41	79 (22.3)	50 (26.2)	0.30	127 (24.9)	34 (17.0)	**0.024**
	Positive	58 (66.7)	505 (79.2)		444 (77.2)	112 (81.2)		151 (75.5)	398 (78.3)		276 (77.7)	141 (73.8)		383 (75.1)	166 (83.0)	
**HER2 status**	Negative	48 (55.2)	514 (79.4)	**<0.001**	437 (75.1)	115 (82.7)	0.056	135 (67.2)	413 (80.4)	**<0.001**	278 (77.7)	153 (78.5)	0.83	375 (73.1)	173 (85.2)	**0.001**
	Positive	39 (44.8)	133 (20.6)		145 (24.9)	24 (17.3)		66 (32.8)	101 (19.6)		80 (22.3)	42 (21.5)		138 (26.9)	30 (14.8)	
**Histological classification**	Invasive ductal	78 (90.7)	524 (81.1)	0.086	478 (82.4)	118 (85.5)	0.69	165 (82.1)	423 (82.6)	0.87	300 (84.0)	158 (81.4)	0.18	432 (84.2)	163 (81.1)	0.24
	Invasive lobular	3 (3.5)	69 (10.7)		55 (9.5)	10 (7.2)		18 (9.0)	51 (10.0)		27 (7.6)	23 (11.9)		40 (7.8)	25 (12.4)	
	Mixed	3 (3.5)	44 (6.8)		37 (6.4)	9 (6.5)		14 (7.0)	31 (6.1)		22 (6.2)	12 (6.2)		34 (6.6)	10 (5.0)	
	Other	2 (2.3)	9 (1.4)		10 (1.7)	1 (0.7)		4 (2.0)	7 (1.4)		8 (2.2)	1 (0.5)		7 (1.4)	3 (1.5)	
**Subtypes**	Luminal A	22 (25.6)	258 (41.5)	**<0.001**	216 (38.2)	60 (44.8)	0.34	74 (37.6)	202 (40.6)	**<0.001**	143 (40.9)	75 (39.9)	0.83	185 (36.9)	89 (46.1)	**0.012**
	Luminal B	14 (16.3)	149 (24.0)		132 (23.4)	31 (23.1)		44 (22.3)	114 (22.9)		80 (22.9)	38 (20.2)		111 (22.1)	48 (24.9)	
	Luminal-HER2	21 (24.4)	83 (13.3)		86 (15.2)	17 (12.7)		30 (15.2)	72 (14.5)		48 (13.7)	25 (13.3)		80 (15.9)	22 (11.4)	
	HER2-enriched	18 (20.9)	47 (7.6)		57 (10.1)	7 (5.2)		36 (18.3)	27 (5.4)		30 (8.6)	17 (9.0)		56 (11.2)	8 (4.1)	
	TNBC	11 (12.8)	85 (13.7)		74 (13.1)	19 (14.2)		13 (6.6)	82 (16.5)		49 (14.0)	33 (17.6)		70 (13.9)	26 (13.5)	

TNBC, triple-negative breast cancer.
Significant p-values are shown in bold.
