# Supplementary material for: Associations of angiogenesis-related proteins with specific prognostic factors, breast cancer subtypes and survival outcome in early-stage breast cancer patients. A Hellenic Cooperative Oncology Group (HeCOG) trial
Source: PLoS One. 2018 Jul 31;13(7):e0200302. doi: 10.1371/journal.pone.0200302 (PMC6067711; doi:10.1371/journal.pone.0200302)
Supplement: S4 Table — (PDF) [file pone.0200302.s004.pdf]

S4 Table. Hazard ratios (95% CIs) estimated from univariate Cox regression for selected clinicopathological parameters.

Parameter	Categories	N of patients	N of events	HR	95% CI	Wald's p
		**DFS**				
**Age (categorical)**	<53.5 vs. ≥53.5	373 vs. 370	126 vs. 129	0.98	0.77-1.25	0.87
**Menopausal status**	Postmenopausal vs.	418 vs. 325	153 vs. 102	1.20	0.93-1.54	0.16
**Surgery**	Breast conserving surgery vs. MRM	259 vs. 482	66 vs. 187	0.59	0.44-0.78	**<0.001**
**Tumor size**	≤2 vs. >2	222 vs. 521	61 vs. 194	0.68	0.51-0.91	**<0.001**
**Number of positive lymph nodes**	0-3 vs. ≥4	354 vs. 389	86 vs. 169	0.46	0.36-0.60	**<0.001**
**ER/PgR status**	Negative vs. Positive	164 vs. 572	62 vs. 189	1.29	0.97-1.71	0.086
**HER2 status**	Negative vs. Positive	573 vs. 172	191 vs. 64	0.82	0.62-1.09	0.18
**Histological grade**	I-II vs. III-IV	371 vs. 368	119 vs. 133	0.82	0.64-1.06	0.13
**Subtypes**	HER2-enriched vs. TNBC	65 vs. 98	24 vs. 38	0.96	0.58-1.60	0.87
	Luminal A vs. TNBC	287 vs. 98	88 vs. 38	0.68	0.47-1.00	**0.048**
	Luminal B vs. TNBC	164 vs. 98	57 vs. 38	0.82	0.54-1.23	0.33
	Luminal-HER2 vs. TNBC	104 vs. 98	39 vs. 38	0.93	0.59-1.45	0.74
**Hormonal therapy**	No vs. Yes	182 vs. 543	67 vs. 187	1.18	0.89-1.56	0.24
**Radiotherapy**	No vs. Yes	171 vs. 546	54 vs. 194	0.88	0.65-1.19	0.39
		**OS**				
**Age (categorical)**	<53.5 vs. ≥53.5	373 vs. 370	98 vs. 101	0.93	0.70-1.22	0.59
**Menopausal status**	Postmenopausal vs. Premenopausal	418 vs. 325	119 vs. 80	1.24	0.94-1.65	0.13
**Surgery**	Breast conserving surgery vs. MRM	259 vs. 482	49 vs. 148	0.56	0.41-0.78	**<0.001**
**Tumor size**	≤2 vs. >2	222 vs. 521	45 vs. 154	0.61	0.44-0.86	**0.004**
**Number of positive lymph nodes**	0-3 vs. ≥4	354 vs. 389	59 vs. 140	0.38	0.28-0.51	**<0.001**
**ER/PgR status**	Negative vs. Positive	164 vs. 572	52 vs. 145	1.39	1.01-1.91	**0.041**
**HER2 status**	Negative vs. Positive	573 vs. 172	149 vs. 51	0.86	0.63-1.18	0.36
**Histological grade**	I-II vs. III-IV	371 vs. 368	93 vs. 104	0.85	0.64-1.13	0.27
**Subtypes**	HER2-enriched vs. TNBC	65 vs. 98	18 vs. 34	0.73	0.41-1.29	0.28
	Luminal A vs. TNBC	287 vs. 98	58 vs. 34	0.50	0.32-0.76	**0.001**
	Luminal B vs. TNBC	164 vs. 98	51 vs. 34	0.80	0.52-1.23	0.31
	Luminal-HER2 vs. TNBC	104 vs. 98	32 vs. 34	0.79	0.49-1.28	0.34
**Hormonal therapy**	No vs. Yes	182 vs. 543	57 vs. 142	1.33	0.98-1.81	0.070
**Radiotherapy**	No vs. Yes	171 vs. 546	39 vs. 154	0.80	0.56-1.13	0.20

N, number; HR, hazard ratio; CI, confidence interval; DFS, disease-free survival; OS, overall survival; MRM, modified radical mastectomy; TNBC, triple-negative breast cancer.
Significant p-values are shown in bold.
